# Ectopic pregnancy risk factors for ART patients undergoing the GnRH antagonist protocol: a retrospective study

**DOI:** 10.1186/s12958-016-0146-0

**Published:** 2016-03-23

**Authors:** A. Weiss, R. Beck-Fruchter, J. Golan, M. Lavee, Y. Geslevich, E. Shalev

**Affiliations:** Department of Obstetrics and Gynecology, Emek Medical Center, Afula, Israel; Rappaport Faculty of Medicine, Technion-Israel Institute of Technology, Haifa, Israel

**Keywords:** In-vitro fertilization, GnRH agonist trigger, Ectopic pregnancy, Oocyte maturation

## Abstract

**Background:**

In-vitro fertilization is a known risk factor for ectopic pregnancies. We sought to establish the risk factors for ectopic pregnancy in GnRH antagonist cycles examining patient and stimulation parameters with an emphasis on ovulation trigger.

**Methods:**

We conducted a retrospective, cohort study of 343 patients undergoing 380 assisted reproductive technology (ART) cycles with the GnRH antagonist protocol and achieving a clinical pregnancy from November 2010 through December 2015.

**Results:**

Significant risk factors for ectopic pregnancy in the univariate analysis included prior Cesarean section (CS), endometriosis, mechanical factor infertility, longer stimulation, elevated estradiol and progesterone levels, GnRH agonist trigger, higher number of oocytes aspirated, and insemination technique. Independent risk factors for ectopic pregnancy in the multivariate analysis included GnRH agonist trigger, higher number of oocytes aspirated, insemination technique, and prior Cesarean section.

**Conclusion:**

Excessive ovarian response, IVF (as opposed to ICSI), prior Cesarean section and GnRH agonist trigger were found to be independent risk factors for ectopic pregnancy. Caution should be exercised before incorporating the GnRH agonist trigger for indications other than preventing OHSS. When excessive ovarian response leads to utilization of GnRH agonist trigger, strategies for preventing ectopic pregnancy, such as a freeze all policy or blastocyst transfer, should be considered. Further studies should elucidate whether adjusting the luteal support can reduce the ectopic pregnancy risk.

## Background

In-vitro fertilization (IVF) is a known risk factor for ectopic pregnancies (EP). In fact the first reported IVF pregnancy was ectopic [1]. During a natural conception, it is assumed that ectopic pregnancy results due to retention of the embryo in the fallopian tube and alterations in the tubal environment allowing early implantation to occur [2]. But after embryo transfer in IVF cycles the embryo must be displaced from the uterus to the fallopian tube for an ectopic pregnancy to occur. In fact ectopic pregnancies have been described in the contralateral fallopian tube after unilateral gamete intra-fallopian transfer (GIFT) [3]. It is therefore likely that reduced endometrial receptivity plays a role in the pathogenesis of ectopic pregnancy.

The incidence of ectopic pregnancies in patients conceived through IVF has been reported to be 2–5 % as opposed to 0.8–2 % in naturally conceived pregnancies [2, 4–6], but this rate has been declining [7]. The ectopic pregnancy rate in the 90’s was between 2.4 to 5.4 % of clinical pregnancies achieved through ART [8–11] with heterotopic pregnancies as high as 15 % of EPs [9, 11]. More recent studies have placed the ectopic pregnancy rate at 1.9–2.1 % for fresh cycles [12–14] which is close to the rate from natural conception pregnancies. The EP rate for frozen/thawed embryo transfers may be even lower [12, 15, 16]. The decline in the ectopic pregnancy rate may be due to a lower number of embryos transferred and/or the reduced frequency in which tubal corrective surgery is utilized. It is also possible that since the introduction of intracytoplasmic sperm injection (ICSI) proportionately more patients in IVF programs suffer from male factor infertility while proportionately fewer suffer from mechanical factor infertility. Finally, the ectopic pregnancy risk may be lower with blastocyst transfer [7, 17].

Fallopian tube pathology is the most well defined risk factor for EP [18], particularly after tubal corrective surgery [19]. Other risk factors which have been reported include smoking [2], endometriosis [20] and prior cesarean section [21]. High estrogen levels during IVF stimulation have also been associated with ectopic pregnancy [22–24] while an association between GnRH agonist trigger and EP has been reported twice [25, 26].

The discovery that GnRH agonist triggering prevents ovarian hyperstimulation syndrome (OHSS) has the potential to change the way IVF is performed. With the risk of OHSS virtually eliminated, we can aim for a large number of embryos increasing the cumulative pregnancy rate per stimulation cycle [27]. Since ovulation trigger with GnRH agonist has become more common and is even being considered in cases with a normal ovarian response [28], it is important to determine whether its use will increase the ectopic pregnancy rate during the fresh cycle.

Indeed, altered endometrial gene expression after GnRH agonist trigger was demonstrated when compared to a control group of oocyte donors triggered with HCG [29]. This transformation was reversed when donors were given a bolus of 1,500 IU hCG 35 h after the GnRH injection, or immediately after ovum pick. This altered gene expression may be a sign of reduced endometrial receptivity and predispose susceptible patients to ectopic pregnancies after receiving a GnRH agonist trigger.

We sought to identify risk factors for ectopic pregnancy by retrospectively reviewing clinical pregnancies attained at our facility through GnRH antagonist ART cycles, particularly focusing on the different final oocyte maturation alternatives, recombinant human chorionic gonadotropin (r-hCG) and GnRH agonist.

## Methods

We accessed a database of all in-vitro fertilization cycles recording patient, treatment and outcome parameters at our facility, collected with the approval of our institutional ethics committee for the purposes of quality control and research. Included were patients whose last menstrual cycle was from November, 2010 (when the database was established), to December, 2015 (the last clinical pregnancy when the data was reviewed).

All couples attending our facility undergo a fertility investigation including a medical, menstrual cycle and obstetrical history, physical examination, a hormone profile study and a sperm analysis. Women are referred to hysterosalpingography (HSG) based upon known risk factors and when a cause for infertility cannot be ascertained from the previous studies.

Male factor infertility was based on the WHO laboratory manual for the examination and processing of human semen [30], polycystic ovary syndrome (PCOS) was diagnosed based upon the Rotterdam criteria [31] and mechanical factor infertility was based on any abnormality of fallopian tube anatomy or pelvic adhesions demonstrated on HSG or laparoscopy. The diagnosis of endometriosis was made only when affirmed surgically.

Included in this study were all women undergoing a GnRH antagonist ART cycles with a sonographically identifiable intrauterine (IUP) or ectopic pregnancy. Patients began gonadotropin stimulation on day three of the menstrual cycle. GnRH antagonists were administered when the leading follicle reached 14 mm and were continued daily up to oocyte maturation triggering. Patients were monitored for follicle growth and endometrial thickness. Estradiol and progesterone levels were typically measured on the day of trigger or one day before. Follicle aspiration was scheduled 34 to 37 h after oocyte maturation trigger [32].

Stimulation protocols were based on the patients expected ovarian response according to prior treatments, age, weight, baseline follicle stimulating hormone (FSH) and antral follicle count. The choice of gonadotropin was flexible and took into account the patients’ preference, insurance coverage, price and compliance with self-administered versus nurse administered compounds. The antagonist used were either Cetrorelix (as acetate) 0.25 mg (Cetrotide, Merck Serono S.A., Switzerland) or Ganirelix 0.25MG (Orgalutran, N.V. Organon, Netherlands). All the participants were triggered with either 250 mcg choriogonadotropin alfa (r-hCG) (Ovitrelle, Merck Serono LTD) or 0.2 mg triptorelin acetate (Decapeptyl, Ferring Pharmaceuticals Israel). Patients who received a dual trigger with both r-hCG and GnRH agonist were not included.

Patients triggered with r-hCG were given luteal support in the form of oral beta estradiol tablets (Estrofem, Novo Nordisk A/D Denmark) 2 mg twice a day and vaginal progesterone suppositories (Endometrin, Ferring Pharmaceuticals Israel) of 100 mg twice a day starting on the day of embryo transfer. Patients were triggered with GnRH agonist if they were deemed at risk of OHSS and were given a more intensive luteal support regimen beginning on the day of follicle aspiration (as opposed to the day of embryo transfer) and consisting of 2 mg beta estradiol tablets and 100 mg progesterone suppositories three times a day each. Alternatively, patients at a lower risk of OHSS, as determined by the physician on the day of oocyte aspiration, were given low doses of hCG as reports have suggested that vaginally administered progesterone may not be sufficient after GnRH agonist trigger [33]. Furthermore, there were patients who received GnRH agonist in an attempt to increase the proportion of mature oocytes (NCT01638026); these patients also received a low dose of hCG.

Embryos were transferred to the uterine cavity 48 or 72 h after oocyte retrieval. The transfer was performed under abdominal ultrasound guidance with either the Cook K-SOFT-5000 catheter, utilizing the optional guide when necessary, or the Wallace Sure-Pro Ultra. Between one to four embryos were transferred, corresponding to the Israel Fertility Association guidelines from 2010 (www.ayala.org.il, Hebrew).

βHCG levels were measured 14 days after embryo transfer. βHCG levels below 5 IU/L were considered negative. Between 5 to 15 IU/L were considered indeterminate and above 15 were considered positive. Patients with indeterminate βHCG levels were considered positive if their βHCG increased after 48 h. All βHCG positive patients had additional βHCG measurements as necessary. Biochemical pregnancies were defined by those patients with declining βHCG levels. These patients were followed until βHCG tested negative and generally did not undergo an ultrasound examination. Patients with increasing or plateau βHCG were followed sonographically. Intrauterine pregnancies were confirmed when a gestational sac with an echoic ring was noted on ultrasound. Sonographic evidence of an ectopic pregnancy included an non-homogenous adnexal mass adjacent to, but separate from the ovary, a mass with an echoic ring surrounding a gestational sac (bagel sign), or a gestational sac with a fetal pole with or without a heartbeat [34, 35]. Patients with biochemical pregnancies or pregnancies of unknown location were not included in the study.

Statistical analysis: Categorical variables are presented as frequencies and percentages. Continuous variables are presented with mean ± standard deviation and [median]. To determine the association between background and treatment parameters to the outcome (IUP versus EP) we used univariate logistic regression utilizing generalized estimating equations methodology (GEE SAS GENMOD procedure). The GENMOD procedure was used for the analysis of correlated data as there were patients who appear more than once in the database. Multivariate logistic regression utilizing GEE was then implemented to ascertain the independent risk factors for the outcome. The estradiol and progesterone levels and endometrial thickness data were adjusted to account for the day they were taken: on the day of oocyte maturation triggering or one day before. Seven values for each variable were not included in the analysis because data was not available on one of those two days. The data were analyzed with SAS 9.2 software (SAS Institute Inc, Cary, NC). A p value < 0.05 was considered to be statistically significant.

## Results

During the study period 840 women underwent 1455 antagonist protocol cycles where at least one fresh embryo was transferred (Table 1). The pregnancy rate among those triggered with GnRH agonist was 26 % vs 31 % among those triggered with rHCG (NS). There were 11 ectopic pregnancies among the 264 women triggered with GnRH agonist (4.17 %) versus 10 ectopic pregnancies among the 1169 women triggered with rHCG (0.86 %) (*P* < 0.001, Fisher’s exact test). There were 22 cycles where both rHCG and GnRH agonist were administered for maturation triggering. We occasionally opt for the dual trigger when patients have a history of empty follicle syndrome [36] or low rate of mature oocytes in prior cycles. These cycles were not included in the analysis. The remaining analysis was done only for the subgroup of patients where it could be ascertained whether the pregnancy was intrauterine or extrauterine.

**Table 1:** Antagonist cycle protocols during the study period

	rHCG trigger	GnRHa trigger	Both	Total
Number	1169	264	22	1455
Age (years)	32.72 ± 6.34 [32]	29.64 ± 5.23 [29]	33.77 ± 5.80 [34]	32.18 ± 6.25 [32]
BMI (kg/m^2^)	25.77 ± 5.42 [24.7]	25.52 ± 5.61 [24.2]	25.52 ± 4.69 [24.3]	25.72 ± 5.44 [24.6]
Duration (years)	2.99 ± 2.58 [2]	3.13 ± 2.55 [2]	3.28 ± 2.38 [2.5]	3.02 ± 2.58 [2]
FSH (IU/L)	7.31 ± 3.02 [6.7]	6.26 ± 2.14 [6.04]	8.06 ± 5.78 [6.25]	7.14 ± 2.96 [6.58]
Oocytes aspirated	7.58 ± 4.50 [7]	14.48 ± 7.95 [13]	7.45 ± 4.06 [8]	8.82 ± 5.91 [8]
Embryos transferred	1.90 ± 0.69 [2]	1.92 ± 0.65 [2]	2.32 ± 1.09 [2]	1.91 ± 0.69 [2]
Pregnant	367 (31 %)	69 (26 %)	4 (18 %)	440 (30 %)
Biochemical	47 (4.0 %)	9 (3.4 %)	0 (0 %)	56 (3.8 %)
IUP	310 (27 %)	49 (19 %)	4 (18 %)	363 (25 %)
EP	10 (0.86 %)	11 (4.17 %)	0 (0 %)	21 (1.44 %)

Included were 343 women undergoing 380 ART cycles and attaining an identifiable pregnancy. There were 359 intrauterine pregnancies and 21 ectopic pregnancies (5.53 % EP rate per cycle resulting in a clinical pregnancy). Among the 21 ectopic pregnancies were an interstitial pregnancy in a patient who had previously undergone a bilateral salpingectomy, a cervical pregnancy and a broad ligament ectopic pregnancy.

The results of the univariate analysis are presented in Table 2. No significant association between ectopic pregnancy and age, infertility duration, BMI, baseline FSH, parity, and prior nonviable pregnancies (ending in miscarriage, ectopic pregnancy or termination of pregnancy) could be found. Patients with an ectopic pregnancy were more likely to have had endometriosis, mechanical factor infertility or a prior Cesarean section. No association was found between the outcomes and whether LH was included in the stimulation protocol, or the total dose of FSH administered. Women with an ectopic pregnancy were more likely to have had conventional IVF rather than ICSI.

**Table 2:** Univariate analysis of patient, treatment and outcome parameters

	Outcome		*P*-value	OR [95 % CI]
	IUP (*N* = 359)	EP (*N* = 21)		
Age (years)	30.93 ± 5.8 [31]	30.74 ± 5.26 [32]	0.7346	0.99 [0.92–1.06]
Duration (years)	2.97 ± 2.43 [2]	3.23 ± 2.39 [2.5]	0.6894	1.03 [0.89–1.2]
BMI (kg/m^2^)	26.68 ± 20.08 [24.61]	25.05 ± 4.94 [22.41]	0.6499	0.98 [0.9–1.07]
FSH (IU/L)	6.96 ± 2.52 [6.59]	6.97 ± 2.62 [6.08]	0.9736	1.003 [0.84–1.19]
Parity	0.47 ± 0.68 [0]	0.62 ± 0.59 [1]	0.3497	1.25 [0.78–1.98]
Prior CS	38 (10.58)	6 (28.57)	0.0275	3.37 [1.14–9.91]
Male factor infertility	244 (67.97)	10 (47.62)	0.0854	0.45 [0.18–1.12]
Endometriosis	11 (3.06)	3 (14.29)	0.0199	4.89 [1.29–18.6]
Mechanical factor	53 (14.76)	8 (38.1)	0.0129	3.53 [1.31–9.53]
Unexplained	46 (12.81)	4 (19.05)	0.4282	1.56 [0.52–4.67]
PCOS	61 (16.99)	3 (14.29)	0.8444	0.88 [0.26–3.01]
IVF insemination	114 (32.02)	15 (71.43)	0.001	5.19 [1.95–13.83]
ICSI insemination	242 (67.98)	6 (28.57)		1
Total FSH dose (IU)	1865.72 ± 938.57 [1675]	2170.24 ± 931.52 [2250]	0.0889	1.03 [0.99–1.08]^a^
LH included in stimulation	131 (36.49)	9 (42.86)	0.6775	1.19 [0.52–2.73]
Stimulation days	8.75 ± 1.84 [9]	9.48 ± 1.81 [9]	0.0273	1.21 [1.02–1.44]
Estradiol (pg/mL)	1470.73 ± 778.02 [1336.4]	2112.88 ± 1302.22 [1679.7]	0.0134	1.08 [1.02–1.14]^a,b^
Progesterone (ng/mL)	0.9 ± 0.4 [0.8]	1.14 ± 0.4 [1.08]	0.0116	2.85 [1.26–6.45]^b^
Endometrial Thickness (mm)	10.25 ± 2.29 [10]	9.22 ± 2.42 [9]	0.0965	0.8 [0.61–1.04] ^b,c^
GnRH agonist trigger	49 (13.65)	11 (52.38)	<.0001	7.55 [3.01–18.96]
r-HCG trigger	310 (86.35)	10 (47.62)		1
Number of oocytes aspirated	8.5 ± 4.97 [8]	12.67 ± 6.62 [11]	0.0033	1.13 [1.04–1.22]
Number of embryos transferred	1.93 ± 0.63 [2]	1.86 ± 0.48 [2]	0.3385	0.72 [0.36–1.42]

Categorical variables are presented as frequencies and percentages. Continuous variables are presented with mean ± standard deviation and [median]

^a^Calculated per 100 unit increase, ^b^adjusted for day measured relative to the day of ovulation trigger (see text), ^c^Calculated per 5 mm increase

Estradiol and progesterone levels were significantly higher in the EP group, as were the number of days of gonadotropin stimulation and the number of oocytes aspirated. Endometrial thickness was not significantly associated with ectopic pregnancy.

Women triggered with GnRH agonist were more likely to have an ectopic pregnancy than those triggered with r-HCG. The odds ratio of having received GnRH agonist for a woman with an ectopic pregnancy was 7.55 with a *P* value < 0.0001. Another way of looking at the data: 60 patients had clinically identifiable pregnancies after GnRH agonist trigger, of those 11 or 18 % had an EP, while among the 320 patients triggered with r-hCG, ten or only 3.1 % had an EP.

Of the sixty patients who were given a GnRH agonist trigger, thirty had luteal support which included hCG and thirty had luteal support regimens which included only progesterone and estradiol supplements. The adjusted odds ratio for ectopic pregnancy for patients without hCG during the luteal phase was 3.16 (CI: 0.63–16.02, *P* = 0.1638). In other words, hCG administered during the luteal phase did not protect patients given GnRH agonist trigger from the ectopic pregnancy outcome.

After multivariate logistic regression analysis (Table 3) factors found to be significantly associated with EP included prior cesarean section, fertilization by IVF and not ICSI, number of oocytes aspirated and GnRH agonist trigger.

**Table 3:** Multivariate analysis

Risk factor	*P*-value	Adj.OR [95 % CI]
Prior CS	0.0089	5.34 [1.52–18.72]
IVF insemination	0.0001	8.83 [2.87–27.13]
GnRH trigger	0.0011	6.37 [2.10–19.33]
No. oocytes aspirated	0.0363	1.09 [1.01–1.19]

Other potential confounders which were tested and found to not correlate with the clinical outcome tested included the type of gonadotropins used (recombinant or urinary), which antagonist was used, day 2 versus day 3 embryo transfer (data for 5 patients who had embryo transfers on days other than 2 or 3 were not included), the identity of the transferring physician, the type of catheter used and the number of embryos transferred.

## Discussion

### Patient Parameters

Mechanical factor infertility, endometriosis, a prior cesarean section and fertilization through IVF without ICSI, were found to associated with ectopic pregnancy. Endometrial thickness was numerically lower in the ectopic pregnancy group but this did not reach statistical significance (*P* = 0.0965) Recently, Rombauts et al. found that a combined endometrial thickness under 9 mm engenders a 4-fold risk of ectopic pregnancy compared with an endometrial thickness over 12 mm [37], which he attributes to the directionality of uterine peristalsis waves.

The association between mechanical factor and ectopic pregnancy is well established, though after multivariate analysis this risk factor was not found to be statistically significant. However, insemination through IVF without ICSI remained significant. Method of insemination is a consequence of patient parameters; the proportion of mechanical factor and endometriosis is higher in IVF patients in comparison to ICSI patients in whom the diagnosis of male infertility is more prevalent. In this respect, the method of insemination is a surrogate parameter for cause of infertility and therefore it was this parameter which remained independent in the multivariate analysis. This association has been described before [8].

Prior Cesarean section was also found to be an independent risk factor for ectopic pregnancy. The reason for this association, which has been described before [21], is unclear though it may be a reflection of reduced endometrial receptivity or the presence of other confounding factors.

### Stimulation parameters

Patients with an excessive ovarian response, as demonstrated with significantly more stimulation days, higher estradiol and progesterone levels and a greater number of oocytes aspirated, were found to be at higher risk for ectopic pregnancy. Multivariate logistic regression indicated that the parameter most closely associated with ectopic pregnancy was the number of oocytes aspirated. GnRH agonist trigger was also found to be associated with ectopic pregnancy. This association remained significant in the multivariate analysis suggesting that GnRH agonist trigger is an independent risk factor for ectopic pregnancy and not just a byproduct of it being administered when ovarian stimulation is excessive. This association between GnRH agonist trigger and EP is of clinical significance since GnRH agonist trigger is being advocated for IVF in general and not only for those patients at risk for OHSS [28].

High estrogen levels during IVF stimulation has already been documented to be associated with ectopic pregnancy [22–24]. Endometrial priming requires adequate estradiol levels for a relatively short duration of time [38], ovarian stimulation leading to supraphysiological steroid production may lead to changes in the endometrium altering its receptivity [39, 40]. Additionally, elevated estrogen levels were found to lead to increased uterine contractions [41]. In a laboratory setting, elevated estrogen levels increased fallopian tube ciliary beat frequency [42]. This combination of increased uterine contractibility with tubal ciliary motion may increase the risk of EP in susceptible patients. In the multivariate analysis the number of oocytes aspirated, and not estradiol levels, remained associated with ectopic pregnancy. Since these two parameters are closely related, it cannot be ruled out that it is indeed the high estrogen levels which are a cause for ectopic pregnancy, but it is feasible that the cause of ectopic pregnancies in hyper-responders is a substance produced by the follicle around the time of the ovulatory trigger other than estrogen. For example, vascular endothelial growth factor A (VEGFA) and prostaglandin E2 (PGE2) both increase dramatically in response to the LH surge [43]. Indeed, these substances have been shown to be elevated in ectopic pregnancies relative to intrauterine pregnancies [44, 45].

### The luteal phase and luteal phase support

There are two other reports of increased ectopic pregnancy after GnRH trigger. Sahin et al., reported an increased rate of ectopic pregnancies in patients triggered with GnRH agonist (6 ectopic pregnancies versus 65 clinical pregnancies or 8.5 %) as compared to those triggered with hCG (5 ectopic pregnancies versus 286 clinical pregnancies or 1.7 %) [25]; they too found that this risk was independent of estrogen levels. Luteal support in this study included 50 mg of progesterone intramuscularly and 5 mg of oral estradiol hemihydrates daily commencing on the day of oocyte aspiration (personal communication).

Sousa et al. reported an increased rate of ectopic pregnancies in patients at risk of OHSS who received the GnRH agonist Buserelin [26]. In their study 4 patients out of 20 (20 %) with a clinical pregnancy after Buserelin trigger had an ectopic pregnancy. This was significantly higher than those receiving HCG for ovulation trigger (1.6 %) who were not at risk for OHSS. Luteal support in this cohort of patients after Buserelin trigger included 600 mg of intravaginal natural-micronized progesterone and one oral tablet of 2 mg oral estradiol commencing on the day of oocyte retrieval.

The additional oral estrogen between follicle aspiration and embryo transfer may be a contributing factor to the increased EP rate due to its effect on uterine contractions and tubal motility. For example, Decleer et al., observed that three of six ectopic pregnancies after frozen-thawed embryo transfer occurred in one year, of a ten year study, after they switched from a natural endometrial preparation protocol to an hormone replacement protocol [12]. Nevertheless, in cycles triggered with GnRH agonist, estrogen support is recommended since the relatively short endogenous LH surge leads to premature corpus luteum demise and may be detrimental to endometrial receptivity [46]. Estradiol levels are significantly lower on the day of oocyte aspiration and embryo transfer when oocyte maturation is triggered with GnRH agonist as opposed to hCG, but they are still relatively high compared to the natural cycle [47–49], therefore it would appear that withholding estradiol between oocyte aspiration and embryo transfer may be done without comprising the luteal phase of these patients.

Engmann et al., introduced the intensive luteal support regimen for women triggered with GnRH agonist [50] consisting of transdermal E2 patches adjusted to maintain an estradiol serum level of 200 pg/mL and intramuscular progesterone of 50 mg daily adjusted to maintain a level above 20 ng/mL. Using this protocol high pregnancy rates were attained but the rate of ectopic pregnancies, if any, was not reported.

An alternative strategy for luteal support is administrating a low dose of HCG at the time of oocyte aspiration but this strategy does not completely prevent OHSS in high risk patients [51, 52]. Orvieto et al., proposed a flexible approach accounting for estradiol levels and the number of oocytes aspirated with an option of HCG luteal support commencing on the day of embryo transfer if there are no signs of early moderate OHSS [53]. In our study, patients who received hCG during the luteal phase were not at a significantly decreased risk of ectopic pregnancy than those that did not. So it is not clear if adjusting the luteal support can alter the ectopic pregnancy risk.

### Embryo transfer technique or day of embryo transfer

Embryo transfer technique has been implicated in the rate of ectopic pregnancies [54, 55]. In our study transfers were performed under abdominal ultrasound guidance with the intention of depositing the embryos in the mid-uterus portion [56]. There was no difference in ectopic pregnancy rate according to the physician conducting the transfer or the transfer catheter used. Also, performing embryo transfer on day 2 or day 3 did not affect the rate of EP.

## Conclusion

We found that ectopic pregnancy was independently associated with, an increased number of oocytes aspirated, prior cesarean section, oocyte insemination through IVF without ICSI, and GnRH trigger.

Insemination by IVF without ICSI is most likely a surrogate parameter encompassing mechanical factor infertility (including cases which remained undiagnosed) and endometriosis and, most likely, does not in itself increase the risk of ectopic pregnancy. The mechanism whereby Cesarean section increases the risk of ectopic pregnancy is unclear but has been described before.

The mechanism whereby a large number of oocytes increases the risk of ectopic pregnancy may be through the closely related elevated estrogen levels or may be mediated through other factors produced by the follicle or corpus luteum.

Finally, we found that GnRH trigger is a risk factor for ectopic pregnancy independent of the other parameters. In our study, 18 % of women triggered with GnRH agonist and with a clinically identifiable pregnancy had an ectopic pregnancy. The corresponding ectopic pregnancy rate was 20 % in the study by Sousa [26] and it was 8.5 % in the study by Sahin [25]. This data is worrisome since GnRH agonist trigger is being utilized more and more, and not only to prevent OHSS.

It is also interesting that of the twenty-one ectopic pregnancies, three were in unusual locations. A recent study found that 28 % of non-tubal ectopic pregnancies were associated with fertility treatment [57] so that these unusual ectopic pregnancies are probably more frequent after ART.

The main drawback of the present study is its retrospective nature and the small number of participants with an ectopic pregnancy, since ectopic pregnancy is a relatively rare outcome of IVF treatment. As a retrospective study based on a database which was collected prospectively, not all parameters of interest were available. While we had data on the parity and gravity of our patients, we were not able to know the number of patients with prior ectopic pregnancies, whether patients smoked or not, whether they had a history of tubal surgery or pelvic Inflammatory Disease. One advantage of this study is that not all patients were given GnRH agonist specifically to prevent OHSS, therefore it was possible to separate the risk associated with hyperstimulaiton versus the risk of the GnRH trigger itself.

The option of triggering oocyte maturity with GnRH agonist and thus largely avoiding OHSS, may entice clinicians to revert to aggressive stimulation protocols. They should be advised that doing so, may place the patient at an increased risk of ectopic pregnancy as well as possibly compromising the quality gametes, embryos and endometrium [58]. When confronted with a patient with risk factors for ectopic pregnancy, particularly high responders, caution is advised. Possible steps which may reduce the risk of EP may include withholding estradiol therapy between oocyte aspiration and embryo transfer to reduce uterine contractions or adopting an intensive luteal support protocol maintaining high levels of progesterone. Further research is needed to determine the optimal luteal support and whether adjusting the luteal support could reduce the ectopic pregnancy rate. Blastocyst transfer may reduce the ectopic pregnancy rate as well [17]. Consideration should be given to refraining from fresh transfer altogether and cryopreserving all embryos [59, 60]; this in accordance with each facilities cryopreservation experience and outcomes. Furthermore, caution should be exercised before adopting GnRH agonist trigger protocols for indications other than the prevention of OHSS.

### Ethics approval

The study was approved by the local institutional review board.

## Abbreviations

aOR: adjusted odds ratio
ART: assisted reproductive technologies
BMI: body mass index
CS: Cesarean section
EP: ectopic pregnancy
FSH: follicle stimulating hormone
GIFT: gamete intra-fallopian transfer
GnRH: gonadotropin releasing hormone
HSG: hysterosalingography
ICSI: intracytoplasmic sperm injection
IUP: intrauterine pregnancy
IVF: in vitro fertilization
LH: luteinizing hormone
OHSS: ovarian hyperstimulation syndrome
PCOS: polycystic ovary syndrome
PGE2: prostaglandin E2
r-hCG: recombinant human chorionic gonadotropin
VEGFA: vascular endothelial growth factor A
